# COMMUNITY-BASED RESEARCH ON GRIEF SUPPORT FOR OLDER ADULTS: FROM EXPLORATION TO IMPLEMENTATION

**DOI:** 10.1093/geroni/igad104.1782

**Published:** 2023-12-21

**Authors:** Lisa Stone-Bury, Rachel Weiskittle

## Abstract

Community-based research has initiated a shift from research existing in an ivory tower to being collected and then implemented for the direct benefit of the public. The social sciences continue to struggle with the balance between rigorous science and feasible interventions, creating a common disconnect between research and practice. This is particularly relevant to grief and bereavement, for which services are often under-utilized among older adults. This symposium will discuss a multidisciplinary team’s application of the Exploration, Preparation, Implementation, and Sustainment (EPIS) framework, describing the development, findings, and service outcomes of a community-based research initiative within a mid-sized city. First, Lisa E. Stone will describe the challenges and successful strategies in the collaborative process of questionnaire development with multidisciplinary community stakeholders. She will also present on the methodology used to attain large community and grief professional samples, including recruitment strategies for adults across the lifespan. Second, Caroline Collins-Pisano will present key age-related findings from the community study, highlighting the lifespan factors that impact grief experiences and utilization of grief resources among older adults. Lastly, Rachel E. Weiskittle will present on the team’s process of implementing data-driven grief interventions, focusing on the translation of community-based findings to multifaceted grief services for older adults. In sum, this symposium presents a community-based application of the EPIS framework that focuses on direct, tailored, and immediate implementation of grief services, a consistent need for the aging population.

